# Cardioprotective Signature of Short-Term Caloric Restriction

**DOI:** 10.1371/journal.pone.0130658

**Published:** 2015-06-22

**Authors:** Hossein Noyan, Omar El-Mounayri, Ruth Isserlin, Sara Arab, Abdul Momen, Henry S. Cheng, Jun Wu, Talat Afroze, Ren-Ke Li, Jason E. Fish, Gary D. Bader, Mansoor Husain

**Affiliations:** 1 Division of Experimental Therapeutics, Toronto General Research Institute, Toronto, Ontario, Canada; 2 Department of Molecular Genetics, University of Toronto, Toronto, Ontario, Canada; 3 Division of Advanced Diagnostics, Toronto General Research Institute, Toronto, Ontario Canada; 4 Department of Laboratory Medicine & Pathobiology, University of Toronto, Toronto, Ontario, Canada; 5 Heart and Stroke Richard Lewar Centre of Excellence in Cardiovascular Research, University of Toronto, Toronto, Ontario, Canada; 6 The Donnelly Centre, University of Toronto, Toronto, Ontario, Canada; 7 Peter Munk Cardiac Centre, University Health Network, Toronto, Ontario, Canada; 8 Department of Medicine, University of Toronto, Toronto, Ontario, Canada; 9 Ted Rogers Centre for Heart Research, University Health Network, Toronto, Ontario, Canada; University of Cincinnati, College of Medicine, UNITED STATES

## Abstract

**Objective:**

To understand the molecular pathways underlying the cardiac preconditioning effect of short-term caloric restriction (CR).

**Background:**

Lifelong CR has been suggested to reduce the incidence of cardiovascular disease through a variety of mechanisms. However, prolonged adherence to a CR life-style is difficult. Here we reveal the pathways that are modulated by short-term CR, which are associated with protection of the mouse heart from ischemia.

**Methods:**

Male 10-12 wk old C57bl/6 mice were randomly assigned to an *ad libitum *(AL) diet with free access to regular chow, or CR, receiving 30% less food for 7 days (d), prior to myocardial infarction (MI) via permanent coronary ligation. At d8, the left ventricles (LV) of AL and CR mice were collected for Western blot, mRNA and microRNA (miR) analyses to identify cardioprotective gene expression signatures. In separate groups, infarct size, cardiac hemodynamics and protein abundance of caspase 3 was measured at d2 post-MI.

**Results:**

This short-term model of CR was associated with cardio-protection, as evidenced by decreased infarct size (18.5±2.4% vs. 26.6±1.7%, N=10/group; P=0.01). mRNA and miR profiles pre-MI (N=5/group) identified genes modulated by short-term CR to be associated with circadian clock, oxidative stress, immune function, apoptosis, metabolism, angiogenesis, cytoskeleton and extracellular matrix (ECM). Western blots pre-MI revealed CR-associated increases in phosphorylated Akt and GSK3ß, reduced levels of phosphorylated AMPK and mitochondrial related proteins PGC-1α, cytochrome C and cyclooxygenase (COX) IV, with no differences in the levels of phosphorylated eNOS or MAPK (ERK1/2; p38). CR regimen was also associated with reduced protein abundance of cleaved caspase 3 in the infarcted heart and improved cardiac function.

## Introduction

High-calorie diets and diminished physical activity are environmental factors believed to contribute to the global epidemic of obesity, metabolic disorders and associated cardiovascular diseases (CVD) [1]. Among numerous proposed strategies to reduce CVD, dietary interventions associated with weight loss seem promising. For example, in experimental animals, even a small change in diet content has been shown to have a significant beneficial effect on CVD [2–4]. In fact, an effective and reproducible intervention for protection of the cardiovascular system is caloric restriction (CR). CR is defined as a reduction of dietary intake, usually 30–40% less than *ad libitum* (AL) levels, without malnutrition [5]. Since 1935, long-term CR has been known to exert an anti-aging effect, extending average and maximum life span and delaying the onset of age-associated pathologies in rat [6].

Several key findings in yeast, worms, flies, and mice point to the existence of diet-responsive genes and signaling molecules which affect health and lifespan (Reviewed in [7]). Recently, health-promoting effects of life-long CR have been demonstrated in non-human primates [8, 9] and to a lesser extent in humans [10, 11], with multiple proposed mechanisms underlying the anti-aging and health-promoting actions of CR [12, 13]. However, the molecular basis of long-term CR is complex, impacting on multiple key signaling molecules and transcription factors involved in metabolism, oxidative stress, inflammation, endoplasmic reticulum stress, mitochondrial apoptosis, increased insulin sensitivity and autophagy (Reviewed in [14, 15]). Such multifaceted actions of CR can potentially result in protection against a broad range of chronic diseases such as diabetes, CVD, neurodegeneration, autoimmune and inflammatory disorders and cancer in humans, similar to what has been shown in rodent models [8, 16–18]. Despite these potential benefits, life-long CR is impractical for the general population and thus of limited clinical relevance. By contrast, emerging data suggest that the beneficial effects of CR may occur rapidly upon initiation [19–21]. Indeed, a *short-term* CR may be a more plausible approach with potential clinical applications, including ischemic preconditioning.

Unfortunately, the literature is devoid of specific definitions for short- (and long-) term CR, and the regimens used have been vastly different with regards to diet composition and duration of CR [21]. The objectives of the current study were two-fold. First, we wanted to test whether a short-term, mild CR before induction of experimental MI can protect the heart from ischemic injury. Second, we wanted to understand the molecular pathways underlying the observed cardiac preconditioning effect of short-term CR using microarray, microRNA and signaling assessments in myocardial tissue. We chose to conduct our investigations in the mouse as this is one of the most commonly studied species in CR pre-clinical trials [22]. We show that a short-term CR (70% of AL) for only 7d prior to MI in mice results in smaller infarct size and improved cardiac function at 2d post-MI and improves survival. This preconditioning effect of short-term CR is associated with a global change in gene expression, which partially resembles that reported for life-long CR [13, 23]. The effect of CR on modulating miR expression in the heart had not previously been examined, nor had the correlations between cDNA and miR analyses been addressed. Our inclusion of specific protein analyses further focuses our findings on known pro-survival pathways responsive to short-term CR. Together, our results propose a feasible and practical nutritional preconditioning regimen that may hold promise for a number of clinical applications.

## Materials and Methods

### Ethics

Protocols were approved by the Animal Care Committee of the Toronto General Hospital (Animal Use Protocol Number 1032.22) in accordance with guidelines of the Canadian Council for Animal Care. This manuscript was prepared according to the ARRIVE (Animal Research: Reporting of In Vivo Experiments) guidelines [24]

### Animals

Male 12 wk-old C57Bl/6 mice were obtained from Charles River (Montreal, PQ, Canada) and maintained in cages (544 cm^2^, 144 PNC Euro Rack System, Allentown Inc.) with corn cob bedding in a conventional facility room on a 12:12 light:dark cycle for at least 2 wk before experimentation. All cages were ventilated and mice were regularly handled by the staff involved in the study. Room temperature was maintained at 21°C with ~50% relative humidity. Mice had free access to food and water during this acclimatization period. Sample size and power calculations were based on our previously published data [25]. Power assessment indicated that a sample size of 5 and 15 per group is required to achieve 95% power for Western blot/microarray/miRNA and infarct size assessments, respectively. Survival from myocardial infarction was not a pre-defined endpoint. These efforts helped to reduce the number of animals used. Animals were randomly assigned to *ad libitum* (AL) diet, stressed AL (sAL, see below) and caloric restriction (CR) groups with no initial differences in body weights. Mice for CR and sAL were individually caged and their food intake was measured everyday between 10–11 am for 1 wk to calculate the average daily food intake. AL mice had free access to food and water and were not disturbed. CR mice were fed 30% less than the calculated mean daily AL food consumption. CR cages were inspected for any remaining food, and transferred to a new cage every day. To control for mouse handling and the possible stress induced by disturbing sleep in CR mice, a sAL group was assigned. sAL group received food *ad libitum*, but were handled similar to CR group at the same times of day. For CR and sAL groups, pre-weighed mouse chow pellets were placed in cages between 10–11 am every day. Body weights were recorded between 10–11 am before and after the 7d study period. For pre-ischemic assessments, animals (n = 5/group/assessment) were sacrificed on 8d (pre-MI), hearts were immediately extracted, washed in sterile phosphate-buffered saline (PBS), with LV free walls dissected, snap frozen in liquid N_2_ and stored at -80°C. Separate groups of AL and CR mice (N = 30/group) underwent permanent left anterior descending (LAD) artery ligation as described previously [25]. Briefly, following induction of anesthesia (see below), mice were intubated and ventilated with a pressure-control ventilator (Kent Scientific). Thorax and pericardium were opened, and the heart was exposed. With the use of a 7.0 silk suture (Deknatel), the LAD was ligated, the chest was closed, and the animal was allowed to recover. Anaesthesia was induced with 4% isoflurane using a Perspex chamber and maintained with 2% isoflurane in 0.5 L/min oxygen. Isoflurane is recommended as the first choice anesthetic in mice and provides rapid induction and safe recovery from anaesthesia with relatively minimal effects on cardiovascular parameters or respiratory rate [26]. With refinement and animal welfare in mind, care was used to minimize pain and suffering of the mice at every pre- and post-surgical stage. For pain management, buperonorphine (0.3 mg/kg), a potent analgesic, was administered subcutaneously immediately following surgery and every 12 h for 72 h. For post-MI infarct measurements (N = 15/group at each time point) including the survival study, animals were monitored daily until humane sacrifice with inhaled CO_2_. The latter was used to avoid the animal stress of handling. There were no observable clinical or behavioral symptoms indicating pain or suffering in mice during 30 days post-MI period. With regards to the survival study, as reported in our earlier publication [25] all deaths in first 2 weeks were due to cardiac rupture. This can result in intra-thoracic hemorrhage and quick death. Inhalation of CO_2_ was used as a humane procedure to sacrifice mice in the end of study period (30d post-MI). Further details regarding the animal experimentation employed in this study can be found in the ***NC3Rs Animal Research*: *Reporting In Vivo Experiments (ARRIVE) Guidelines Checklist*** provided in Supplementary Information.

### Infarct size

At 2d post-MI, mice were harvested, hearts extracted, and atria were discarded before sectioning the LV with a mouse heart slicer matrix (Zivic Insruments, Pittsburgh, PA, USA). Each 2mm thick slice was incubated in 2% triphenyl tetrazolium chloride (TTC, Sigma-Aldrich), at 37°C to differentiate viable from infarcted myocardium, followed by fixation in 4% paraformaldehyde for 30 min at room temperature. Both sides of the slices were then scanned and the infarct size reported as percent infarct, (area of infarct: total LV surface area)*100, determined by computer planimetry using Image J (version 1.40) NIH software.

### Cardiac hemodynamics

To investigate hemodynamic effects of CR, LV pressure-volume loops were obtained on sAL and CR mice at the baseline (after 7 days of sAL and CR regimens and before MI, D0) and 2d post MI (D2) (N = 5/group) as described previously [27].

### Western blot

For assessment of signaling pathways, apoptosis, mitochondrial markers and LC3B expression known to be affected by long-term CR, protein extracts from LV at 7d of sAL and CR regimens (N = 5/group) were run on denaturing gels, blotted and hybridized with primary Abs overnight. HRP-conjugated 2° Abs were visualized with chemiluminescence. Blots were then scanned by a BioRad GS-800 calibrated densitometer and quantified using Quantity One software (Ver. 4.6.1.). 1° and 2° Abs used for Western blot are listed in Table A in S1 File.

### Microarray

Data from the microarray studies described in this paper represent N = 5/group and have been deposited in the NCBI/GEO database (Accession Number GSE68646). These data are in compliance with the Minimal Information About a Microarray Experiment (http://www.mged.org/Workgroups/MIAME/miame.html). The global gene-expression pattern of AL and CR mice (three replicates in each group) were compared using mouse MOE 430.2 Affymetrix Gene Chip Array. Statistical analysis and visualization was performed using Partek Genomics Suite 6.5 and GX11 software. Data analysis and visualization was performed using Partek version 6.5 [Partek Inc] and GX11 [Agilent] software. In a previous publication, we showed that pre-treatment of mice with a weight-reducing dose (comparable to CR group) of the glucagon like peptide-1 agonist, liraglutide, for 7 d resulted in similar cardioprotection post-MI, as demonstrated by reduced incidence of cardiac rupture [25]. Therefore, in addition to the aforementioned groups, we also performed microarray analysis of the LV tissues isolated from liraglutide pre-treated mice (N = 5). This was performed to assess if similar levels of weight loss due to CR or drug treatment (both resulting in similar degrees of cardioprotection) were associated with similar effects on gene expression.

### qRT-PCR

For RNA extraction and qRT-PCR of mRNA transcripts, total RNA from the LV free wall of AL, sAL and CR mice was extracted with TRIZOL reagent (Life Technologies) and tested for purity (UV absorption ratio, A260/A280 > 1.8). DNase I (Fermentas) treatment was performed at room temperature for 30 min. RNA was reverse transcribed using the qScript cDNA SuperMix (Quanta Biosciences). Final primer concentrations used were 0.2 μM with 60°C annealing temperature for all primers. Standard curves were generated for all primers and samples were run in triplicate. These analyses were performed on Roche LightCycler 480 System as per the manufacturer’s instructions, and normalized to expression of the housekeeping gene Gapdh (Mouse) Primers used are listed in Table B in S1 File.

### miR expression and validation

QuantiMiR qRT-PCR (Systems Biosciences, Mountain View, CA) assays were performed on total RNA (800 ng) extracted using TRIZOL reagent, from AL and CR mice (N = 3/group), as per manufacturer’s instructions. Briefly, poly(A) tailing and cDNA synthesis were performed using the QuantiMiR Reverse Transcription Kit. qRT-PCR was performed with the reverse-transcribed miR, and 2X SYBR GREEN qPCR Master Mix buffer. miR assay primers for the full complement of 709 individual mouse miR, along with a universal reversed primer, was provided in the miRNome miR Profiler Kit, and qPCR was performed on the Roche LightCycler 480 System. Three endogenous controls, mmu-U6, RNU43 & U1, were run on each plate. Data analysis was performed by means of ΔΔCt calculation software provided by the manufacturer. To validate the differential expression of miR as identified by profiling, total RNA from AL and CR mice (N = 5/group) was extracted as above. cDNA was synthesized using either the qScriptmR cDNA Synthesis Kit (Quanta Biosciences) or the Taqman miR Reverse Transcription kit (Applied Biosystems) from 100 and 10 ng of total RNA respectively. For PerfeCTa SYBR Green SuperMix qPCR, primers corresponding with the exact sequences of each individual miR (Integrated DNA Technologies) (Table B in S1 File) and the universal reverse primer supplied by the manufacturer (Quanta Biosciences) were used and samples were analyzed in triplicate. miR expression was normalized to endogenous control U6. For the Taqman system, AL and CR cDNA samples were diluted 1:3 and 2 μL was used for qPCR using Taqman Primer sets (PN442795) for hsa-miR-24 (000402), hsa-miR-92a (000431), hsa-miR-92b (007028_mat), hsa-miR-491 (001038) and U6 snRNA (001973) (Applied Biosystems). Real-time PCR was conducted on 2 μL of cDNA in triplicate using Roche 480 Probes Master Mix for Taqman and analyzed with a Roche Lightcycler 480®. Data was normalized to U6 snRNA using the ΔΔCt method.

### Pathway enrichment analysis

Normalized gene expression for CR and AL samples were used in Gene Set Enrichment Analysis (GSEA) [28] to uncover pathways and processes enriched with differentially regulated genes. Each gene was scored for differential expression using the signal to noise statistic (calculated by GSEA). Pathway sets were collated from a broad set of pathway databases and Gene Ontology annotations (http://download.baderlab.org/EM_Genesets/, downloaded April 15, 2013). Enriched pathways (P<0.005 and FDR<0.05) were visualized in Cytoscape (version 2.8.3) [29] as an Enrichment map [30] where nodes are gene sets (pathways) and lines (edges) indicate the overlap between gene sets (calculated using an overlap coefficient, cut-off 0.5) which brings together terms that share a large proportion of genes and helps reduce the redundancy that exists in functional annotation. The thicker the edge, the higher the overlap between pathways. Enrichment maps were further annotated with miR gene sets (defined by Targetscan 6.2 [31]) using the post analysis feature of the enrichment map software to highlight gene sets that contain statistically significant (hyper-geometric P<0.05) percentage of miR targets.

### Statistical analyses

**Fig 1:**
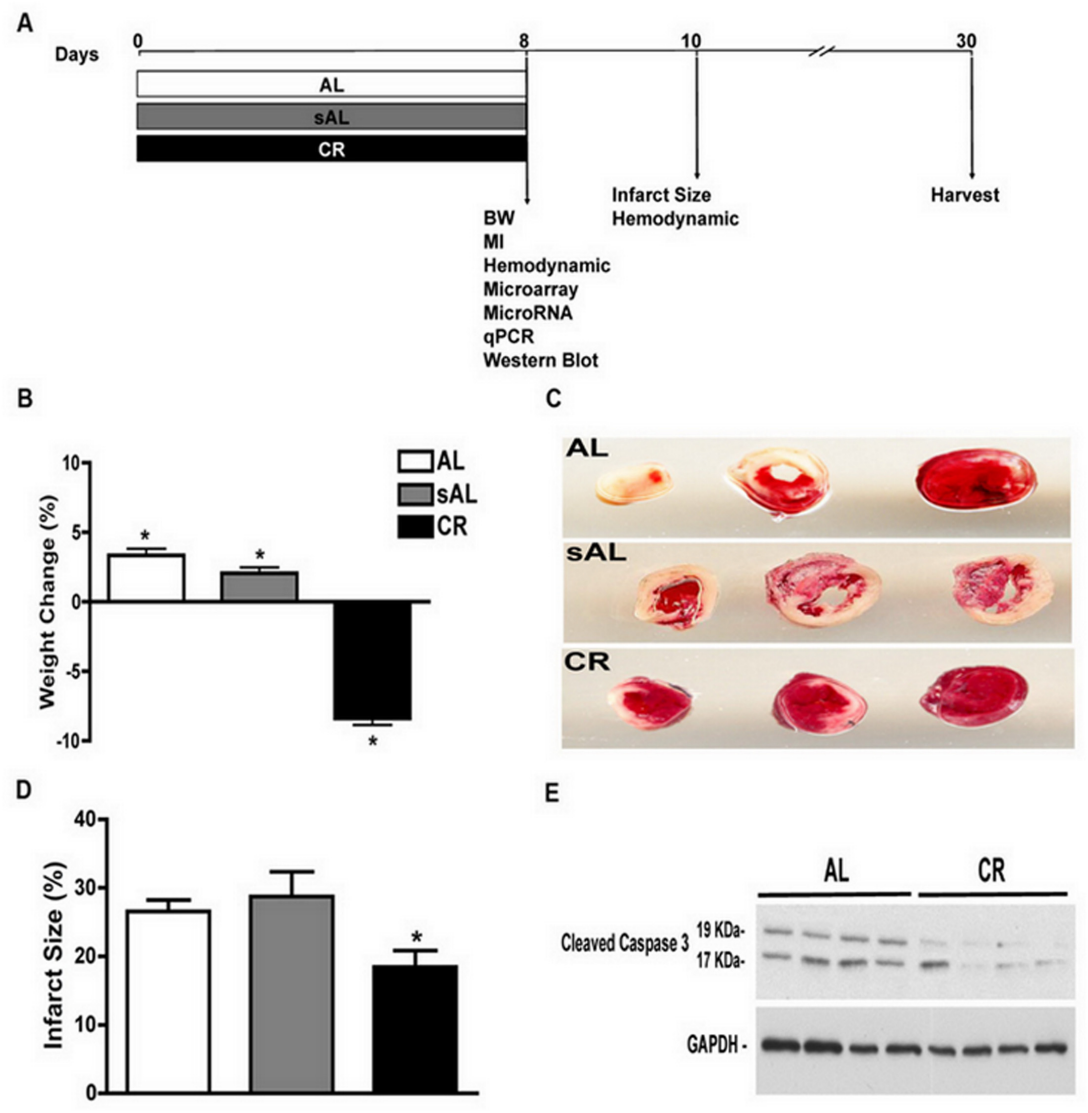
Short-term CR is associated with mild weight loss and reduces infarct size following permanent LAD ligation. **(A)** Schematic of the protocol adopted for short-term caloric restriction (CR) with control groups AL (*ad libitum*) and sAL (stressed *ad libitum*), (**B)** Percent weight change over 7d shows an 8.5% weight loss in CR, with 8.6% and 6.7% weight gain in AL and sAL, respectively. (**C)** Representative TTC stained cardiac slices and (**D)** quantitative analysis of infarct size at 2 d post LAD ligation showing reduced infarct size in the CR group compared to AL and sAL controls (18.45±2.41 *vs*. 26.55±1.69 and 28.7±3.6; respectively, N = 15/group, 1-way ANOVA and Tukey post hoc test, P = 0.01). (**E)** Representative Western blots for cleaved caspase-3 performed on total lysates from hearts obtained d2 post-MI reveal reduced induction of this apoptosis marker in CR-treated animals.

All data are expressed as mean ± SE. Un-paired Student’s *t* test was used to compare data shown in Fig 1. Statistical analysis and visualization of microarray data was performed using Partek version 6.3 [Partek Inc] and GX11 [Agilent] software. qPCR data shown are mean ± SEM. Statistical analyses of qPCR data were performed by Student's unpaired t-test or One-way ANOVA followed by Tukey's for multiple comparisons. Analyses were performed on Graphpad Prism v5.0 (GraphPad Software, Inc., La Jolla, CA, USA).

## Results

### Short-term CR induces weight loss and reduces infarct size and apoptosis in a mouse permanent LAD ligation model of MI

A schematic representation of the experiments performed is shown in Fig 1A. Mice were randomly assigned to AL, sAL and short-term CR regimens, and there were no baseline differences in body weight between these groups (P = NS). Short-term CR (7d) resulted in weight loss (-2.0±0.7 g, P = 0.02, N = 15) as compared to weight gain observed in the AL (+0.85 ±0.4 g, P = 0.04) and sAL groups (+0.67 ±0.6 g, P = 0.0009) (N = 15/group) (Fig 1B). Analysis of infarct size based on TTC-stained heart slices at 2d post-MI revealed reduced infarct size in CR mice as compared to AL and sAL controls (18.45±2.41 *vs*. 26.55±1.69 and 28.7±3.6; respectively, N = 15/group, P = 0.01; Fig 1C and 1D). Cleaved caspase 3, a marker of apoptosis was also down-regulated in the infarct region of the CR mice compared to the AL group (Fig 1E). In a separate group of mice intended for later time point analysis, we also observed improved survival post-MI in CR as compared to AL controls (N = 15/group, *P* = 0.001) (Fig A in S2 File). Together, these results highlight a cardioprotective effect of short-term (7d) CR in a permanent LAD ligation model of MI.

### Short-term CR improved cardiac function post-MI

**Fig 2:**
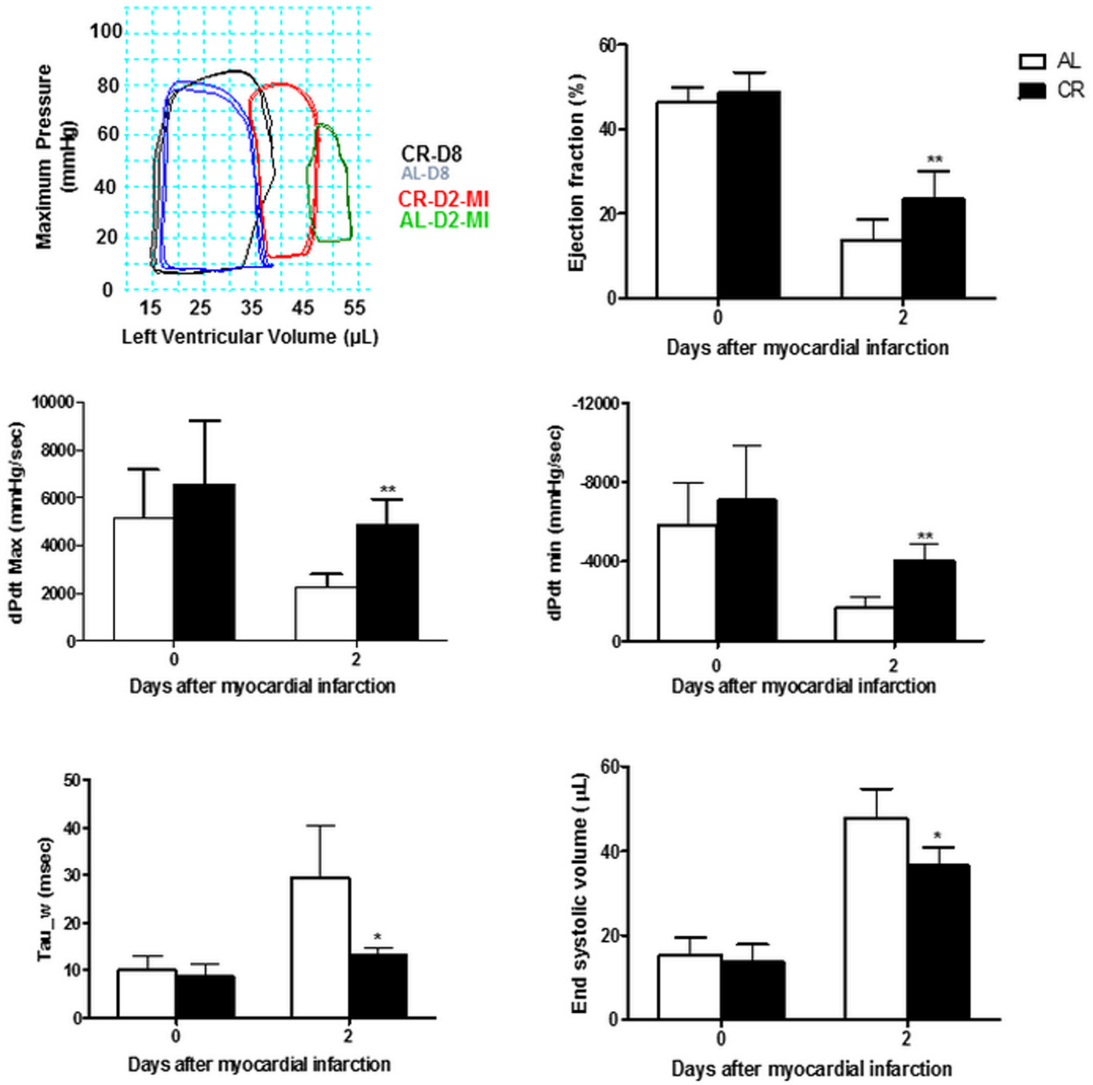
Short-term CR improved cardiac function post-MI. **(A)** Representative steady state pressure-volume (PV) loop recordings in the two treatment groups pre- (D8 of diet = D0-MI) and 2 days post-MI (D2-MI). (**B–F)** Grouped results of PV analyses reveal differences in systolic (ejection fraction, dP/dt-max and end-systolic volume) and diastolic function (dP/dtmin and Tau) in favor of CR- *vs*. sAL-treated mice (*P<0.05 and **P<0.01 respectively).

Invasive pressure-volume measurements (Fig 2) showed reduced systolic function with lower dP/dt max (first derivative of pressure during isovolumic contraction) and ejection fraction as well as larger end-systolic volume in AL *vs*. CR mice at 2d post-MI. Reduced first derivative of pressure during isovolumic relaxation (dP/dt min) coupled with prolonged time constant of isovolumic relaxation (tau) suggested abnormalities in myocardial energetics and stiffness in AL *vs* CR mice after severe ischemic insult. Importantly, there were no differences between the sAL and CR groups in any of the hemodynamic parameters prior to MI (0d).

### Short-term CR is associated with altered cardiac gene expression

**Fig 3:**
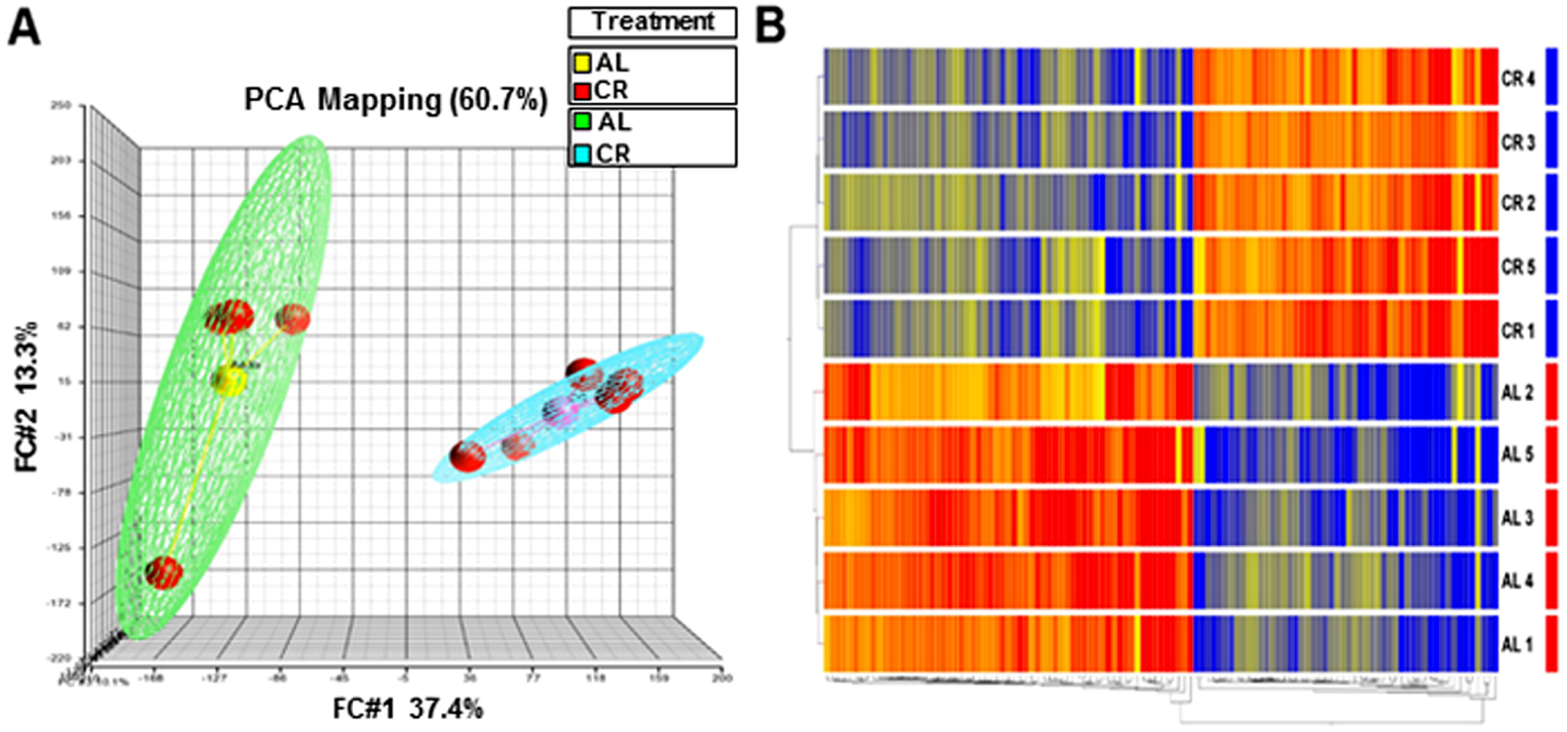
Short-term CR is associated with robust changes in LV gene expression profile. Principal Component Analysis (PCA) depicting overall data. (**A)** PCA representation of replicates to visualize overall clustering of the microarray gene expression profiles of CR (blue mesh) and Ad lib (green mesh) containing 5 replicate samples (red) in each group. PCA depicts complete separation of overall pattern in CR vs. Ad lib (AL) samples, (**B)** The genes differentially expressed (upregulated or downregulated in the CR relative to AL) are clustered according to treatment group. The color gradient (red, up-regulation; green, down-regulation) represents normalized gene expression of each gene in all of the samples listed on the right side of each row. The dendrogram on the left shows the grouping of samples based on the similarity of gene expressions amongst them. Samples were clearly grouped according to CR treatment.

**Fig 4:**
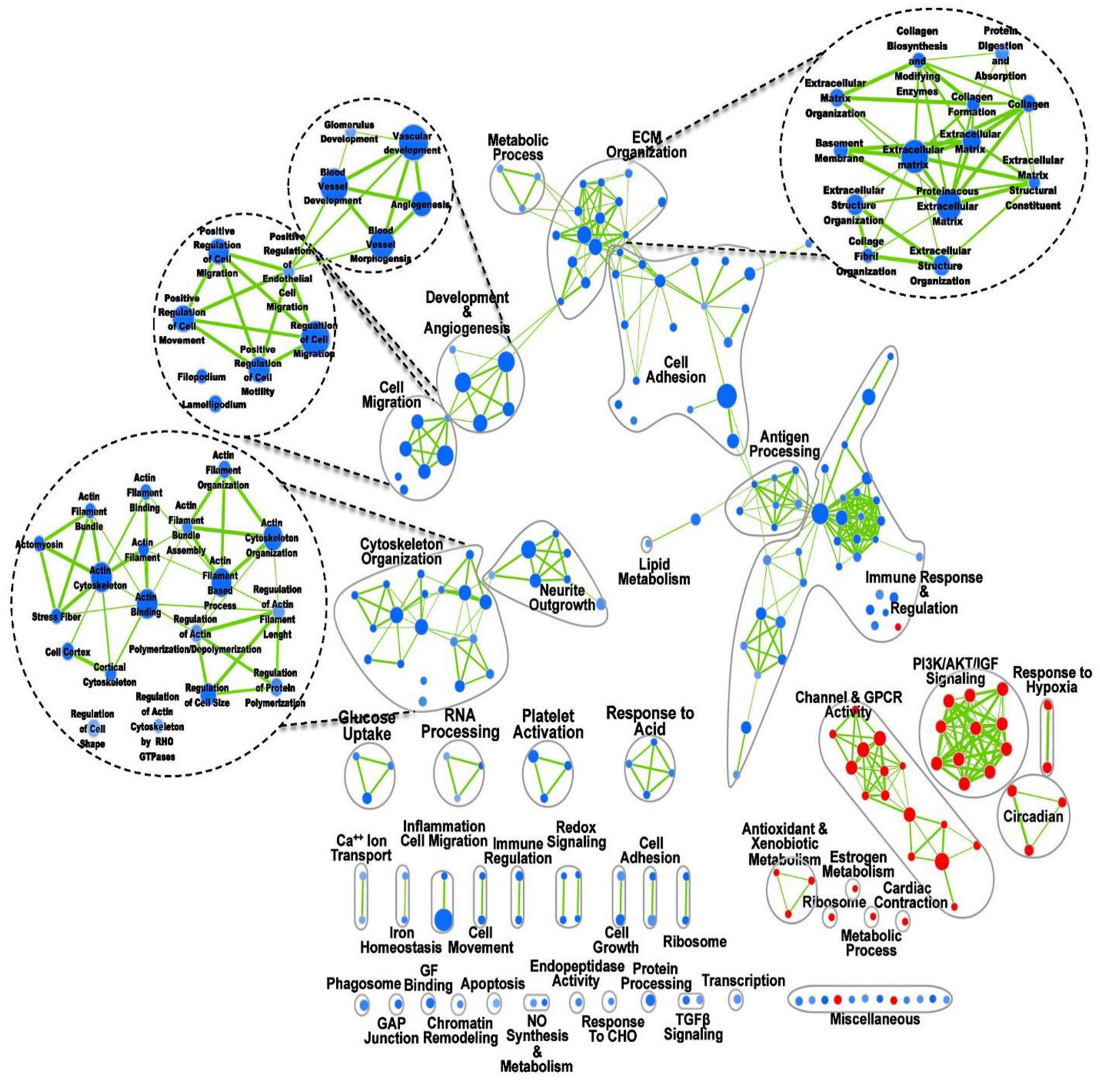
Pathway analysis of short-term CR *vs*. *ad libitum* (AL) groups. The map displays the enriched pathways (blue nodes are downregulated and red nodes are upregulated) in short-term CR versus AL control. Clusters of functionally related sets were manually circled and assigned a label. Dashed circles represent zoom in of clusters of pathways of interest. Nodes represent enriched pathways and edges the similarity between those pathways calculated based on an overlap statistic > 0.5. Enrichments were filtered by p-value < 0.01, FDR < 0.05. Thickness of edges represents the degree of overlap between adjacent pathway nodes.

Microarray analysis was conducted to examine the global LV gene expression profile underlying the demonstrated preconditioning effect of short-term CR. The gene-expression patterns of CR and AL mice were compared using MOE 430.2 Affymetrix Gene Chip Array. A total of 6977 transcripts were significantly regulated (unpaired T-test corrected P≤0.05 false discovery rate (FDR) Benjamini-Hochberg), with 115 genes showing ≥2-fold up-or down-regulation in CR *vs*. AL. Both principal component analysis (PCA) and hierarchical clustering (heat maps) demonstrated clear differences in global gene expression between the two groups (Fig 3A and 3B). We also conducted quantitative PCR (qPCR) validation of several genes that were differentially regulated in the array of CR *vs*. AL (Fig. B in S2 File). To identify pathways and processes that were enriched with differentially regulated genes in CR *vs*. AL, we next conducted a GSEA (Fig 4). Pathways associated with ECM remodeling, angiogenesis, cell morphology, migration and immune response were down-regulated in CR. Interestingly, there was high enrichment in pathways associated with anti-oxidative stress, circadian rhythmicity and biological clock, which have been previously described in life-long CR studies [13, 23, 32]. Our results demonstrate that short-term CR for only 7d promotes global changes in cardiac gene expression, resembling in part what has been described in long-term CR.

We also compared the CR-induced gene expression data in the present study with those obtained from the hearts of mice pre-treated with a weight-reducing dose of the glucagon-like peptide (GLP)-1 agonist, liraglutide (Lira) (200 ug/kg, twice daily, i.p. for 7 days prior to MI) [25]. This was undertaken to compare and contrast the gene expression profiles of two distinct methods of weight reduction (CR *vs*. Lira), in an attempt to understand the mechanisms underlying the reduced (a) incidence of cardiac rupture observed post-MI in Lira-treated animals [25], and (b) infarct size at 2d post-MI observed in CR-treated animals (which was not observed in the Lira study) [25]. Intriguingly, despite similar degrees of weight loss, Lira-treated mice showed minimal changes in gene expression as compared to AL groups (Fig. C in S2 File).

### Sleep disruption and handling stress has no effect on gene expression profile of CR

**Fig 5:**
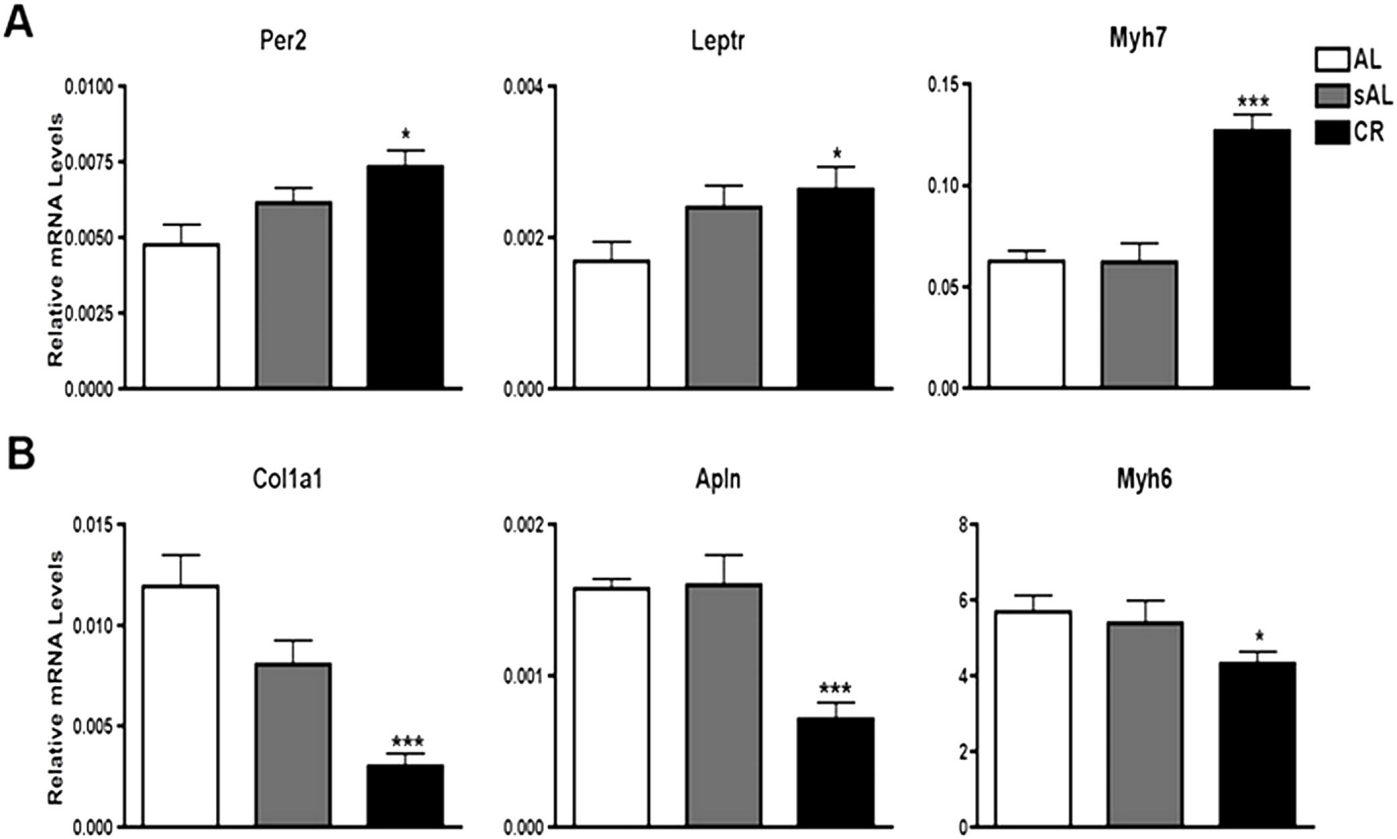
Stressed mice (sAL) did not display a similar gene expression pattern to CR treatment. qPCR analysis of the expression levels of Per2, Leptr, Myh7, Col1a1, Apln and Myh6 in AL, sAL and CR groups following one week of dietary regimens. No changes in gene expression were detected in AL and sAL control groups indicating that stress due to sleep disruption in sAL mice has no effect on the expression of CR-responsive genes. mRNA levels were normalized to the housekeeping gene Gapdh.

Expression levels of selected mRNA up-regulated (Per2, leptr and Myh7) and down-regulated (Col1a1, Apln and Myh6) by short-term CR were comparable between AL and sAL groups (One-way ANOVA with Tukey post-hoc test, P<0.05; Fig 5A and 5B). This shows that the modulation of gene expression observed in the CR group was not related to the stress of sleep disruption or food handling. Similar infarct size in the AL and sAL groups further supported the specific cardioprotective effects of CR observed in this study (Fig 1D).

### Short-term CR is associated with a unique cardiac miR signature

**Fig 6:**
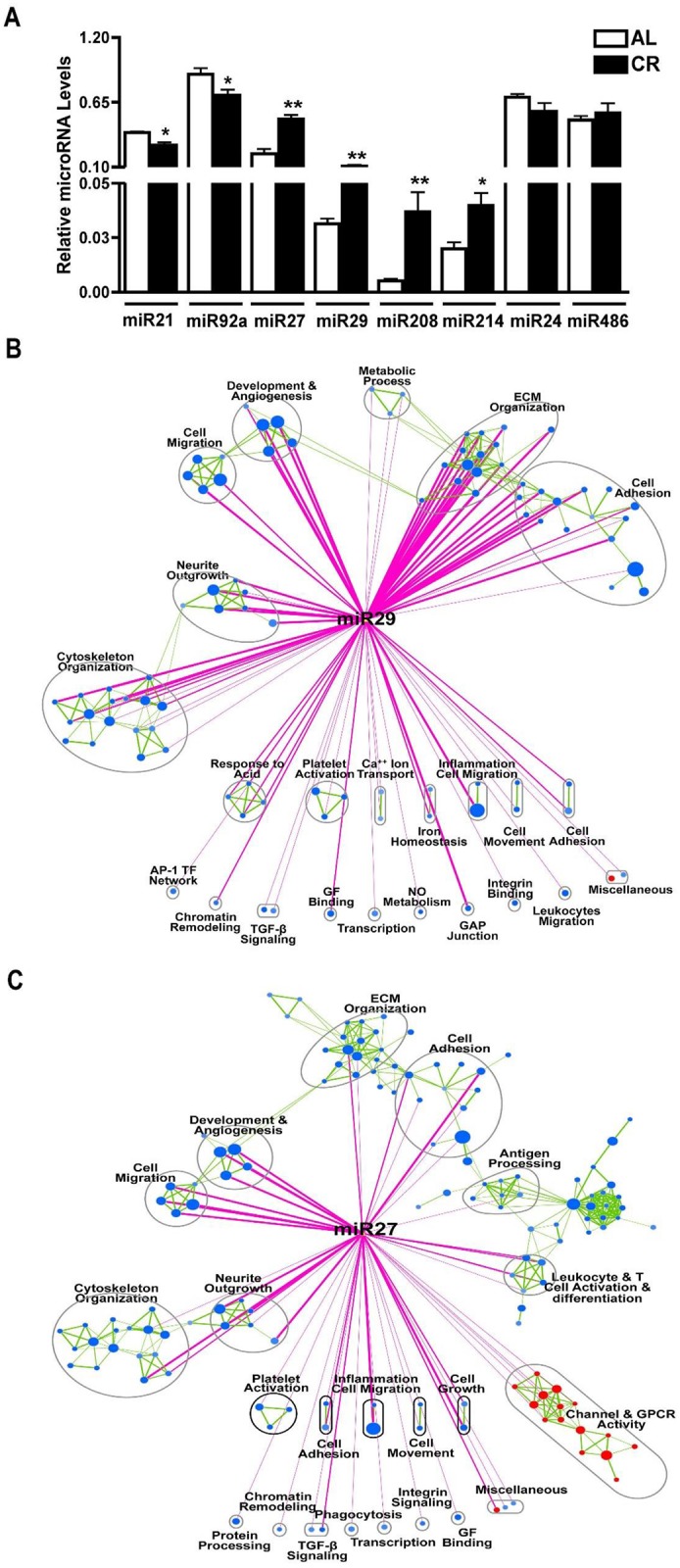
Short-term CR is associated with a complex miR expression profile. **(A)** qPCR validation of selected miRs from the microRNA array showing significant down-regulation of miR-21 and miR-92a and significant up-regulation of miR-27, miR-29, miR-208 and miR-214 in CR compared to Ad lib. miR-24 and miR-486 were used as negative controls. miR levels were normalized to U6. (**B & C)** Enrichment Map of CR vs Ad lib. Nodes represent enriched pathways and edges the similarity between those pathways calculated based on an overlap statistic > 0.5. Enrichments were filtered by p-value < 0.01, FDR < 0.05. The enrichment map was further annotated using a post analysis using mir-29 and mir-27 gene sets to highlight enriched pathways (blue or red nodes) that contain a statistically significant amount of B) miR-27 or C) mir-29 targets (as calculated using the hypergeometric distribution, p-value < 0.05). Post analysis edges are colored in pink, and enrichment map edges are colored in green.

To determine whether the 7d of CR used in our study is accompanied by differential regulation of miR, qPCR arrays were used to determine the expression of 709 distinct miRs in the LV of CR and AL mice. miR array analysis revealed 18 miR that were up-regulated and 24 miR that were down-regulated by CR (Table 1). To validate findings from the miR array, 6 miRs (miR-21, -92a, -27, -29, -208 and -214) were tested by qPCR using SYBR Green or Taqman systems. As shown in Fig 6A, the levels of miR-21 and miR-92a were down-regulated in CR as compared to AL mice (-1.085±0.2465, P<0.01; and -1.798±0.6679, P<0.02; fold change respectively, N = 3). In addition, miR-27, miR-29, miR-208 and miR-214 were significantly up-regulated in CR as compared to AL groups (+2.969±0.5318, P<0.05; +7.483±1.084, P<0.002; +2.483±0.9468, P<0.009; and +2.003±0.5865, P<0.02; fold change respectively, N = 3) (Fig 6A). Our qPCR results demonstrated no change in the level of miR-24 and miR-486 in CR *vs*. AL groups (N = 3), again confirming results of the miR-array (Fig 6A). Together, these data indicate a strong correlation between miR-array and qPCR. We then annotated the enrichment map with the predicted targets of two miR with the highest fold change in CR (miR-27 and -29) to highlight gene sets that are significantly modulated (hyper-geometric P<0.05) as a percentage of miR targets (Fig 6B and 6C). Both miR-27 and miR-29 showed overlapping targets in pathways associated with ECM organization, angiogenesis, cell migration, adhesion and cytoskeletal organization. We also validated by qPCR the expression levels of known targets of three miR (miR-27, -29 and -214), which were up-regulated in the CR group. Of note, miR-214, which has previously been shown to reduce Ca^2+^ overload-induced cardiomyocyte death in an ischemia-reperfusion injury model [33], was up-regulated in CR mice. Up- and down-regulation of miR in the present study correlated well with mRNA levels of their known and predicted gene targets as demonstrated by microarray analysis and qRT-PCR results (Fig. B in S2 File). For example, down-regulation of several distinct miR (-34a, -199a, -181a/b and -204), which have been shown to target Sirt1 [34, 35] was associated with up-regulation of the mRNA level of Sirt1 in CR (Fig. A in S2 File). Similarly, while miR-133a and miR-181 were down-regulated, the mRNA level of their target, pro-survival gene Mcl1 [36, 37] was up-regulated in the CR heart (Fig. A in S2 File). On the other hand, up-regulation of miR-214 in the CR group was associated with down-regulation of mRNA levels of its known target genes, Ncx1 and Camk2d (P< 0.01 and P<0.02 respectively) in the hearts of CR as compared to AL mice (Panel B in Fig. B in S2 File). In addition, mRNA levels of Col1a1, Mmp2, and Itg6, known targets of miR-29 (Up-regulated in the CR mice) [38], were also down-regulated (P<0.001, P<0.003 and P<0.0001 respectively) in CR hearts (Panel B in Fig. B in S2 File). Also, mRNA levels of the tumor suppressor gene Fbxw7, a known target of miR-27 [39], was down-regulated (P<0.01) in the hearts of CR *vs*. AL mice (Panel B in Fig. B in S2 File). These results show, for the first time, that short-term CR for only 7d mediates a cardioprotective gene profile that includes specific miR and their downstream targets. For further details, a large listing of genes regulated by CR is provided in Table C in S1 File.

**Table 1:** Fold changes in heart miR expression levels in short-term CR *vs*. AL.

Upregulated	Fold change	Downregulated	Fold change
mmu-mir-27a	1.68	let7e	-5.63
mmu-mir-29b	2.35	let7f	-4.35
mmu-mir-106b	2.34	mmu-mir-1	-9.38
mmu-mir-140	4.66	mmu-mir-15a	-3.12
mmu-mir-208	7.12	mmu-mir-21	-17.34
mmu-mir-214	3.03	mmu-mir-22	-3.36
mmu-mir-340-3p	7.05	mmu-mir-23a	-8.82
mmu-mir-409-3p	3.04	mmu-mir-34b-5p	-4.29
mmu-mir-468	3.57	mmu-mir-34c	-6.09
mmu-mir-669h-5p	3.31	mmu-mir-92a	-0.43
mmu-mir-669j	3.03	mmu-mir-127	-6.88
mmu-mir-709	4.15	mmu-mir-133a	-3.42
mmu-mir-805	3.92	mmu-mir-181d	-3.09
mmu-mir-1937a	6.26	mmu-mir-188-3p	-7.27
mmu-mir-1954	5.14	mmu-mir-188-5p	-18.76
mmu-mir-2138	5.14	mmu-mir-199a-5p	-3.57
mmu-mir-2142	8.23	mmu-mir-204	-5.37
mmu-mir-2144	5.63	mmu-mir-297b-5p	-13.48
		mmu-mir-323-5p	-8.00
		mmu-mir-324-3p	-6.66
		mmu-mir-343	-4.88
		mmu-mir-449a	-4.82
		mmu-mir-532-3p	-5.85
		mmu-mir-720	-13.02
		mmu-mir-135	-7.22
		mmu-mir-1190	-5.56
		mmu-mir-2132	-12.93
		mmu-mir-2133	-11.74

### Short-term CR modulates major signaling pathways in the heart

**Fig 7:**
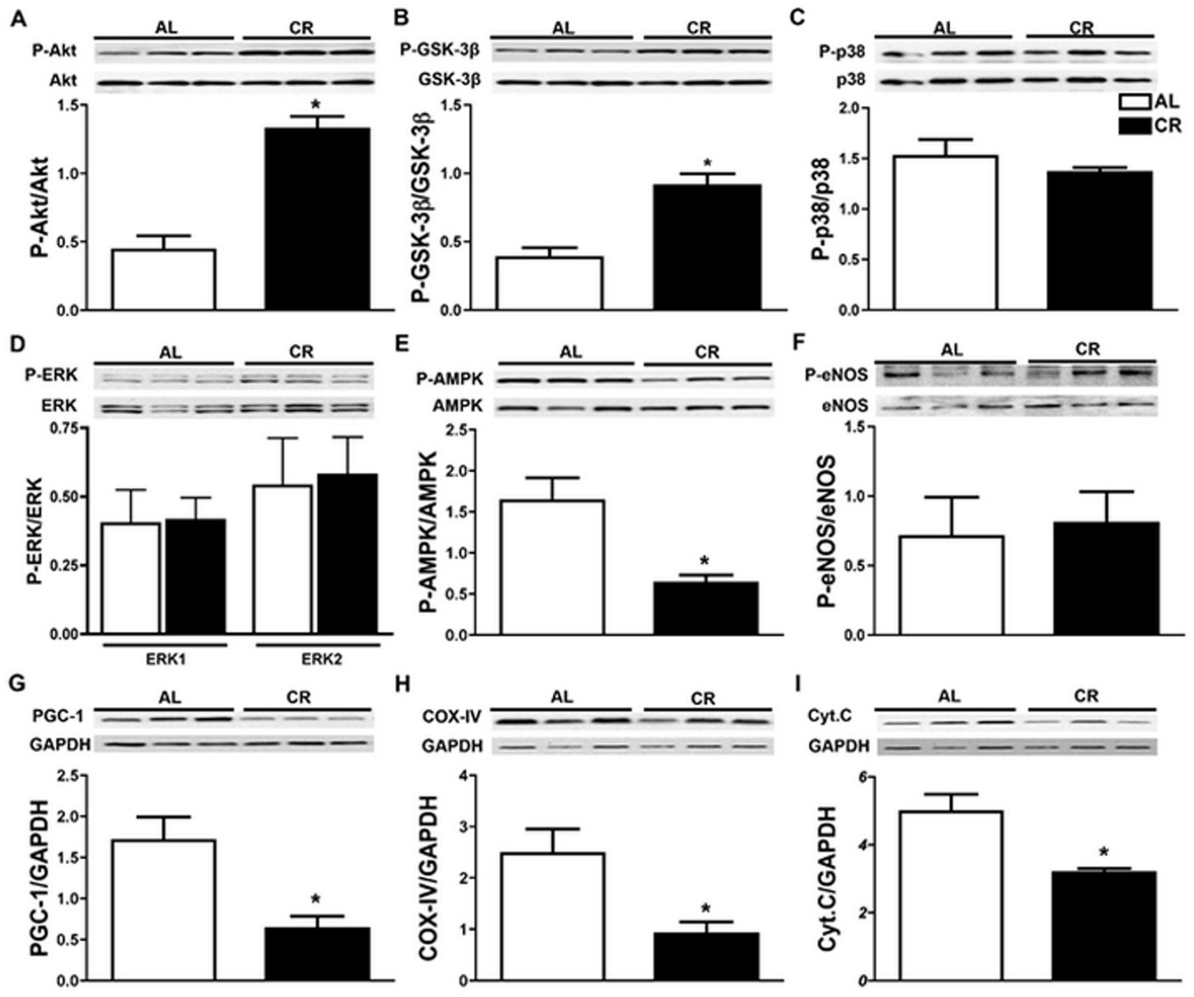
Short-term CR induces known pro-survival pathways. Western blots analysis of the left ventricular protein extracts after 7 days of caloric restriction depicting significant (**A)** decrease in AMPK phosphorylation, (**B** & **C**) increase in phosphorylation of AKT and its downstream target GSK-3β compared to AL group. Phosphorylation levels of ERK, p38 MAPK and eNOS (**D-F**) were comparable between the CR and AL mice. Abundance of the mitochondrial proteins PGC-1α, Cytochrome C and COX-IV were significantly reduced in the CR group compared to the AL mice.

Next, the phosphorylation (P~) status of signaling pathways previously shown to be involved in cardiac protection such as AMPK [40], Akt/PKB [41], GSK3β [42], ERK and p38 MAPK [43] were examined by Western blot. Densitometry demonstrated a significant increase in P~AKT (pAkt/Akt: AL 0.4±0.1 *vs*. CR 1.3±0.1, P<0.05; 3.25±0.1 fold change) and its downstream target GSK3β (P~GSK3β/GSK3β: AL 0.4±0.05 *vs*. CR 0.9±0.1, P<0.05; 2.25±0.1 fold change) (Fig 7A and 7B). By contrast, the phosphorylation status of two members of the MAPK signaling pathway, namely p38 and ERK, were not changed in response to CR (Fig 7C and 7D). We also observed a significant reduction in phosphorylation of the α-subunit of the AMP-activated protein kinase (AMPKα) in CR as compared to AL mice (P~AMPK/AMPK: P AL 1.64±0.3 *vs*. CR 0.6±0.1, P<0.05; -2.75±0.3 fold) (Fig 7E). By examining down-stream targets of AMPK, such as eNOS and mitochondrial markers, PGC-1, cytochrome c, and COX IV, we found no difference in both total and phosphorylated eNOS levels in the hearts of CR *vs*. AL mice (Fig 7F), but did find decreased protein abundance of the mitochondrial markers noted above (PGC-1α: AL 1.7±0.3 *vs*. CR 0.6±0.1; -2.85±0.3 fold change; COX-IV: AL 2.5±0.5 *vs*. CR 0.9±0.2, -2.85±0.3 fold change; Cytochorome C: AL 5±0.5 *vs*. CR 3.2±0.1, P<0.05, -1.6±0.1 fold change) (Fig 7G–7I). In addition, protein abundance of autophagic marker LC3B-II were reduced in the hearts of CR mice (AL 0.3±0.02 *vs*. CR 0.07±0.006, P<0.05, -4.3±0.1 fold change) (Panel A in Fig. A in S2 File). Importantly, sAL mice showed no change in Akt phosphorylation (Panel B in Fig. B in S2 File) indicating that the increased Akt phosphorylation observed following CR is specifically related to the dietary manipulation rather than the stress of animal handling. Together, these results demonstrate that short-term CR modulates known signaling pathways implicated in cardio-protection, mitochondrial function and biogenesis.

## Discussion

In the current study we have demonstrated that short-term (7d) CR protects the mouse heart against ischemic injury with reduced infarct size and cleaved caspase-3 induction at 2d post-MI, with improved survival and cardiac hemodynamics. Our data suggest that this occurs via CR-induced activation of known pro-survival signaling pathways, *i*.*e*. Akt/GSK3β axis, and induction of a gene signature involved in the regulation of cardiac ECM and cytoskeletal organization, cell migration and adhesion, oxidative stress, inflammation, biological clock, apoptosis and autophagy. Differential gene expression and pathway analysis suggest a multi-mechanistic effect of short-term CR, potentially stemming from a complex miR expression profile in the preconditioned LV. Differentially regulated miRs target many of the genes identified in the microarray analysis, contributing to the distinct enrichment pathway map of CR.

Although cardioprotective actions of CR have been previously described, the species, strain, age, duration, percent and type of restricted calories and CR protocols, as well as cardiac injury models adopted have varied significantly (see [44] for a Review). For example, in rat *i)* CR (60% of AL) for 10 months was associated with less cardiac inflammation in an ischemia-reperfusion (I/R) model *in vivo* [45], with *ii)* eight months of CR (55% of AL) also protecting the isolated heart against global ischemia and reperfusion [46], and *iii)* 2 wk of CR (90% of AL) followed by another 2 wk of more robust CR (65% of AL) [47], or *iv)* six months of CR (10% of AL for 2 wk followed by 65% of AL for 24 wk) [48], and *v)* very mild CR (90% of AL) for 2 wk followed by 24 wk of more aggressive CR (60% of AL) [49] also protecting isolated hearts from ischemia-reperfusion (I/R) injury.

Previous studies conducted on long-term CR have suggested attenuation of oxidative stress, mitochondrial dysfunction and inflammation, and a favorable modulation of energy metabolism, apoptosis and autophagy [12, 14, 50–53] to be among cellular mechanisms that increase life-span and promote cardioprotection. While translational significance of the aforementioned CR protocols is uncertain, our CR regimen offers a feasible and practical preconditioning strategy which may afford important clinical applications such as surgical stresses, inflammatory diseases, chemotherapy and insulin resistance (as suggested by Robertson and Mitchel [21]). Indeed, our very short-term CR protocol promotes preconditioning effects with robust cardioprotection similar to long-term CR regiments. This is important since in practice it is almost impossible for individuals to adhere to a long-term CR.

Life-long CR has been shown to protect aged myocardium partially through reversing age-associated reductions in AMPK phosphorylation [54]. AMPK serves as an energy sensor, is a key regulator of mitochondrial biogenesis and autophagy, and is involved in cardioprotective mechanisms [40, 55]. Thus, life-long CR may enhance the energy status of the aged heart by maintaining or inducing activation of AMPK. In addition, life-long CR is associated with maintaining or increasing expression of genes targeted by AMPK such as those involved in mitochondrial function and biogenesis [56] and autophagy [57]. However, in our study, we noted that short-term CR regimen we employed actually *reduced* cardiac AMPK phosphorylation, and *decreased* protein abundance of mitochondrial proteins involved in biogenesis (PGC-1α, COXIV and cytochrome C). In addition, although autophagic flux had not been examined, unlike life-long CR, protein expression of autophagy marker LC3B (and LC3B to GAPDH ratio) [58] was also reduced (Fig. D in S2 File). The mechanism of short-term CR-induced reduction of cardiac AMPK phosphorylation and down-regulation of mitochondrial and autophagic proteins remains to be elucidated. We can only speculate on how the moderate and short-term energy deficiency induced by CR results in a cardioprotective effect, which has been suggested in other metabolic models [59, 60]. Altogether, the effect of short-term CR on selected signaling and target molecules associated with key metabolic pathways seems to be distinct from that of life-long CR.

Life-long CR is also associated with *decreased* cardiac P~Akt levels [57], which correlate with the down-regulation of the insulin/IGF pathway, playing a key role in promoting health and longevity in several experimental model organisms [61–64] including humans [11]. By contrast, our short-term CR model resulted in a robust *increase* in P~Akt (activation) and its down-stream target GSK3β (inactivation), which are known to confer cardioprotection as prosurvival kinases [65–67]. Akt is a serine/threonine protein kinase which regulates cardiac growth, myocardial angiogenesis, glucose metabolism, and cell death in cardiac myocytes [41]. The level and duration of Akt activity determine the balance between its beneficial and harmful effects. While chronic (age-associated) up-regulation of Akt phosphorylation may result in adverse cardiac hypertrophy, a short-term increase in Akt phosphorylation (as observed in the present study) is shown to be cytoprotective and physiologic [68].

Similar to life-long CR [23], short-term CR resulted in increased cardiac expression of the biological clock genes, Per2 and Per1. Clock genes are reproducibly modulated by CR regimens [23]. Emerging data implicate clock genes in many aspects of normal physiology such as energy balance, glucose homeostasis, and diseases especially in the cardiovascular system, playing important roles in innate and adaptive immunity [69–74]. Therefore, clock genes may also be involved in the beneficial effects of CR, considering they coordinate and modulate energy metabolism, transcription, signaling, growth, metabolism and contractile function in cardiac cells [70]. In fact, modulation of cardiomyocyte circadian clock and subsequent tolerance to ischemia-reperfusion has been suggested and has been shown to be time-of-day-dependent [69]. In addition, a recent study in an ischemia-reperfusion model showed that Per2 knockout mice had larger infarcts and greater inflammatory cell infiltration in the infarct zone as compared to wild-type controls [75]. Indeed, stabilization of Per2 has been suggested as a promising strategy for treating myocardial ischemia [76]. Thus, up-regulation of cardiac clock genes (Per1 and Per2, as a result of preconditioning) may provide a mechanism to protect the heart against ischemic and cytotoxic insults, possibly through adapting cellular metabolism to harsh environmental conditions.

Previous network analysis of whole-genome microarray data showed down-regulated genes by long-term CR were highly connected and located in dense genetic network regions but up-regulated genes were weakly connected and positioned in sparse network regions [32]. Similar to life-long CR [23], genes down-regulated by short-term CR displayed high connectivity suggesting that those genes are disproportionately located in dense network regions. Down-regulated genes in the LV were associated with immunity, collagen, ECM, cell adhesion, and insulin signaling while up-regulated genes were mostly associated with circadian rhythm, anti-apoptotic, anti-inflammatory and anti-oxidant functions. Despite being of short duration, the result of microarray analysis in the present study suggests that many aspects are similar to life-long CR. As previously shown for the mouse liver [77], short-term CR can reproduce the most important beneficial aspects of lifelong CR in the heart. It also implies that CR confers its complex beneficial effects through collective interactions of many genes and proteins. In addition, we show that very short-term CR modulates the expression levels of several miRs, which are known to play critical roles in cardiac development [78], function [79, 80] and cardioprotection [33]. Most importantly we show that both miR-27 and miR-29 have overlapping targets associated with ECM organization, angiogenesis, cell migration, cell adhesion and cytoskeletal organization. As such, our data raise the possibility that genes involved in ECM remodeling, cell morphology and angiogenesis, which have not been well explored in the context of cardioprotecion and preconditioning, may be intricately involved in the cardioprotection of CR.

Similar to life-long CR [23], short-term CR response in the myocardium included upregulation of enzymes and molecules like methalothionein 1 and 2 which are stress-response proteins with anti-apoptotic and anti-oxidative actions [81], which elicit cardioprotective roles in ischemia/reperfusion injury [82]. Additionally, genes involved in regulation of cardiac ECM were among those down-regulated by CR. Molecules like matrix metalloproteinases (MMPs), tissue inhibitors of MMPs (TIMPs) and miR are present and/or embedded in the matrix [83]. Cardiac ECM is important for synchronized beating of cardiac myocytes resulting in maintenance of the contraction relaxation cycle, lineage specification and self-renewal of stem cells, biochemical stability of growth factors, facilitating signal transduction and processes such as angiogenesis, fibrosis, autophagy, and inducing apoptosis (Reviewed in [84, 85]). Thus, modulation of ECM genes in the heart by short-term CR may have significant functional consequences which remain to be determined.

We previously showed that pretreatment with a weight-reducing dose of a glucagon-like peptide-1 (GLP-1) agonist, liraglutide is associated with activation of Akt/GSK3β axis, reduction of cardiac rupture post-MI and improved survival with no effect on infarct size at 2d post MI [25]. Interestingly, although both our previous liraglutide pretreatment study and the present short-term CR experiments (both 7d-long regimens) induced comparable weight loss and activated similar prosurvival kinase (Akt/GSK3β) pathways, the global gene expression signature and the cardioprotective outcome were distinct and more robust with short-term CR (see Fig. C in S2 File). These findings suggest that CR and liraglutide have different mechanisms of actions, with short-term CR having a more potent cardioprotective effect as defined by infarct size post-MI.

In conclusion, the current study shows that a simple, short-term CR for only 7d can provide meaningful protection from a model of permanent ischemia (without reperfusion injury) in the mouse. The multifaceted effects of this short-term CR strategy suggests that its conferred cardioprotective actions are due to several overlapping and interdependent signaling, metabolic, genetic, physiologic, and cellular mechanisms. Identifying molecular mechanisms by which short-term fasting affects cells and organ systems may lead to the development of novel prophylactic, preventive and therapeutic interventions for variety of disorders [86]. In addition, if key genes and/or microRNA targets of CR can be identified then it may be possible to metabolically activate CR even under *ad libitum* feeding using gene or miRNA targeting as shown in *Caenorhabditis elegans* (miR-80 [87]). Currently, such a strategy represents an aspirational frontier in the fight against obesity and other metabolic diseases, and may also enable healthier aging. Finally, it may be possible to achieve the striking cardiovascular benefits of short-term CR in clinical practice by incorporating such a strategy through short-term dietary regimens or the use of CR mimetics.

### Limitations

This small exploratory pre-clinical study has been performed in relatively young mice, which have previously served as excellent small animal models for short- and long-term CR and myocardial ischemia. However, they have several physiological, metabolic and behavioral differences from older animals, in which cardiovascular diseases are more likely to occur; and have specific anatomical, physiological, immunological, metabolic and behavioral differences compared with humans. As such, the translational implications of the current study need to be confirmed in large animal models using older age animals, and in early-phase clinical trials in humans.

## Supporting Information

S1 File **Table A** provides a detailed list of primary and secondary antibodies used for this study. **Table B** contains primer sequences used for real time RT-PCR. **Table C** (included as a separate excel file) lists up- and down-regulated genes in CR vs. AL. Further details regarding the animal experimentation employed in this study are provided through the Animal Research: Reporting In Vivo Experiments (ARRIVE) guidelines checklist.
(ZIP)

S2 File **Fig. A**. Survival post-MI was significantly higher in the CR mice compared to AL controls as demonstrated by Kaplan-Meier survival curves (N = 15, *P* = 0.001). **Fig. B**: qPCR validation of differentially regulated genes upregulated (Panel A) and downregulated (Panel B) genes in CR vs. AL from the DNA microarray analysis. The mRNA levels were normalized to the housekeeping gene Gapdh. **Fig. C**: Hierarchical clustering representation of the genes differentially expressed (genes upregulated or downregulated 1.2 fold in the heart samples of Liraglutide and CR group relative to Ad lib). The color gradient (red, up-regulation; bleu, down-regulation) depicts normalized gene expression of all of the samples. Panel A: the heat-map of gene expression depicts a more similar expression pattern in Liraglutide and AL conditions with an opposite pattern of expression in CR. Panel B: Principal Component Analysis (PCA) representation of replicate microarray gene expression profiles of heart samples from CR, Liraglutide, and AL mice (green, blue, and red symbols, respectively). PCA to visualize overall clustering of the microarray data showed that the transcriptional profiles were reproducible with more similar expression pattern in Liraglutide and Ad lib conditions and discrete from CR. **Fig. D**: Western blot assessment of LC3 and p-Akt/Akt expression. Protein abundance of autophagy marker LC3A/BII is significantly reduced (P = 0.001) in the LV of CR mice pre-MI (Panel A). AL and sAL regimens had no effect on the phosphorylation status of Akt, while CR resulted in increased phosho-Akt levels (Panel B).
(ZIP)
