# Supplementary material for: Cardioprotective Signature of Short-Term Caloric Restriction
Source: PLoS One. 2015 Jun 22;10(6):e0130658. doi: 10.1371/journal.pone.0130658 (PMC4476723; doi:10.1371/journal.pone.0130658)
Supplement: S1 File — Table A provides a detailed list of primary and secondary antibodies used for this study. Table B contains primer sequences used for real time RT-PCR. Table C (included as a separate excel file) lists up- and down-regulated genes in CR vs. AL. Further details regarding the animal experimentation employed in this study are provided through the Animal Research: Reporting In Vivo Experiments (ARRIVE) guidelines checklist. (ZIP) [file pone.0130658.s001.zip › S1_File-Noyan_et_al. FINAL2.docx]

**Supporting Information**

**S1 File.** **Table A** provides a detailed list of primary and secondary antibodies used for this study. **Table B** contains primer sequences used for real time RT-PCR**. Table C** (included as a separate excel file) lists up- and down-regulated genes in CR vs. AL. Further details regarding the animal experimentation employed in this study are provided through the Animal Research: Reporting In Vivo Experiments (ARRIVE) guidelines checklist.

**Table A: Primary & Secondary Antibodies**

| **Antibody (Cat #)** | **Source** | **Supplier** | **Dilution** | **Application(s)** |
| --- | --- | --- | --- | --- |
| **Akt (9272)** | Rab PC | Cell Signaling Technol. | 1:1000 | WB |
| **P-Akt (Ser473) (9271)** | Rab PC | Cell Signaling Technol. | 1:1000 | WB |
| **AMPK (2603)** | Rab MC | Cell Signaling Technol. | 1:1000 | WB |
| **P-AMPK (Thr172) (2535)** | Rab MC | Cell Signaling Technol. | 1:1000 | WB |
| **Cleaved Caspase-3 (Asp175)(9661)** | Rab PC | Cell Signaling Technol. | 1:500 | WB |
| **COX IV (4844)** | Rab PC | Cell Signaling Technol. | 1:1000 | WB |
| **Cytochrome C (sc-7159)** | Rab PC | Santa Cruz Biotechnol. | 1:200 &1:2000 | IF & WB |
| **eNOS (9672)** | Rab PC | Cell Signaling Technol. | 1:1000 | WB |
| **P-eNOS (Ser1177) (9571)** | Rab PC | Cell Signaling Technol. | 1:500 | WB |
| **ERK1/2 (4695)** | Rab MC | Cell Signaling Technol. | 1:1000 | WB |
| **P-ERK ½ (Thr202/Tyr204) (9101)** | Rab PC | Cell Signaling Technol. | 1:1000 | WB |
| **GAPDH (sc-25778)** | Rab PC | Santa Cruz Biotechnol. | 1:10000 | WB |
| **GSK3β (9315)** | Rab PC | Cell Signaling Technol. | 1:1000 | WB |
| **P-GSK3β (Ser9) (9336)** | Rab PC | Cell Signaling Technol. | 1:1000 | WB |
| **LC3 A/B (4108)** | Rab PC | Cell Signaling Technol. | 1 : 4000 | WB |
| **PGC-1α (sc-13067)** | Rab PC | Santa Cruz Biotechnol. | 1:2000 | WB |
| **p38MAPK (9212)** | Rab PC | Cell Signaling Technol. | 1:1000 | WB |
| **P-p38MAPK (Thr180/Tyr182) (9211)** | Rab PC | Cell Signaling Technol. | 1:1000 | WB |
| **Anti-Rab IgG-HRP conjugate (170-6515)** | Goat | BioRad | 1:10000 | WB |

Table B: Primer sequences used for real time RT-PCR

| Gene | Forward primer | Reverse primer |
| --- | --- | --- |
| Apln | CCAGAACTTCGAGGACTGGAC | GCCAAATAGATGTGAGGGTTCC |
| Aplnr | TCTGAGGTTCAAGCCAATCC | GCTTCCAGTCTGCGTAGTCG |
| Cat | TGGTTTTCACTGACGAGATGG | CCTTTGCCTTGGAGTATCTGG |
| Ccnd1 | CTGCCGAGAAGTTGTGCATC | GAAATCGTGGGGAGTCATGG |
| Col1a1 | AATGGCATCCCAGGAGAAAAG | GTTTTCCATCATTGCCTGGTC |
| Crtc2 | CTACAGCAGTTGCCCTCTCAG | CAGATTCCCCAAGCTGAAGTC |
| **Erg1** | CAACCCTATGAGCACCTGACC | GCGGCCAGTATAGGTGATGG |
| Fbxw7 | ACTGGAGAATTTTGGCTGAGG | TCCATGGGCTGTGTATGAAAC |
| Fam107a | AGAGACTGAACCAGCTGGAAAAC | AGGGAGCTCTACAGTGCTCTTTC |
| Foxo3 | GTGCGCTGTGTGCCCTACT | TCTGAACGCGCATGAAGC |
| Itgb6 | TGCTCCTCAAAGCTTGGTTC | TCCATAGAGGCGGAGAGGTC |
| Gadd45b | CGCTCTGCAGATTCACTTCAC | ATGACAGTTCGTGACCAGGAG |
| Gstm1 | CGTATGTTTGAGCCCAAGTGC | CGTGTAGCAAGGGCCTACTTG |
| Gstt2 | AGACGGAAGCTTCGTGTTGAC | CCGAAAGTACCACGGATGTTG |
| Lepr | CTTTGGGAATGAGCAAGGTC | TCAAGTCCCCTTTCATCCAG |
| Mcl1 | AAGCTCCAGCCACCAAACTAC | CCACAATCCTGTAGCCACTTTC |
| Mmp2 | GTTGGCAGTGCAATACCTGAAC | ATCTGGGTTGCCACATCTTG |
| Mt1 | CTGCGCCTGCAAGAACTG | GTCCGATACTATTTACACGTGGTG |
| Mt2 | CCGATCTCTCGTCGATCTTC | CATTTGCATTGTTTGCATTTG |
| Myh6 | GCCAACCTGGAGAAAGTGTC | GCTGGGTGTAGGAGAGCTTG |
| Myh7 | GAGACGGAGAATGGCAAGAC | GATCATCCAGGAAGCGTAGC |
| Per1 | CGGATTGTCTATATTTCGGAGCA | TGGGCAGTCGAGATGGTGTA |
| Per2 | CACAGCCCGAGCTAGAGAC | GTGAGGCCCAACTTCTGAAGA |
| Per3 | CTCCGCCCCTACAGTCAGAA | CCGGAACCTGTCAACGTAGT |
| Pink1 | GTGGAATATCTCGGCAGGTTC | GAAAACCCGGATGATGTTAGG |
| Ncx1 | AAGCTCCAGCCACCAAACTAC | CCACAATCCTGTAGCCACTTTC |
| Postn | CGCAGTGATGCCTATTGACC | TCTCCAGTCCTCTGCGAATG |
| Thrb1 | GTCCTCAGTGATGCGCTTATG | CTGCAGGTCACTGTGAGAAGG |
| Tfrc | AGGCAGACCTTGCACTCTTTG | CTGCAGCAGCTCTTGAGATTG |
| Sirt1 | CAGCTGATGAGCCACTTGC | TGGTCTCACTTTCAGAGAAGATCC |
| Gapdh | ACTGTGGATGGCCCCTCTGG | TGACCTTGCCCACAGCCTTG |

Apln, Apelin; Aplnr, Apelin receptor; Cat, Catalase; Ccnd1, Cyclin D1; Col1a1, Procollagen TypeIII alpha1; Crtc2, CREB regulated transcription coactivator 2; Erg1, Early growth response 1; Fbxw7, F-box and WD-40 domain protein 7; Fam107a, Family with sequence similarity 107, member A; Foxo3, Forkhead box O3; Itgb6, Integrin beta 6; Gadd45b, Growth arrest and DNA-damage-inducible 45 beta; Gstm1, Glutathione S-transferase, mu 1; Gstt2, Glutathione S-transferase, theta 2; Lepr, Leptin Receptor; Mcl1, Myeloid cell leukemia sequence 1; Mmp2, Matrix metalloproteinase 2; Mt1, metallothionein 1; Mt2, metallothionein 2; Myh6, Myosin heavy polypeptide 6; Myh7, Myosin heavy polypeptide 7; Per1, Period Homolog 1; Per2, Period Homolog 2; Per3, Period Homolog 3; Pink1, PTEN induced putative kinase 1; Ncx1, Slc8a1 solute carrier family 8 (sodium/calcium exchanger); Postn, Periostin, osteoblast specific factor; Thrb1, Thyroid hormone receptor beta, transcript variant 1; Tfrc, Transferin Receptor; Sirt1, Sirtuin 1 ; Gapdh, Glyceraldehyde-3-phosphate dehydrogenase.
