# Supplementary figures and images for: Cardioprotective Signature of Short-Term Caloric Restriction
Source: PLoS One. 2015 Jun 22;10(6):e0130658. doi: 10.1371/journal.pone.0130658 (PMC4476723; doi:10.1371/journal.pone.0130658)

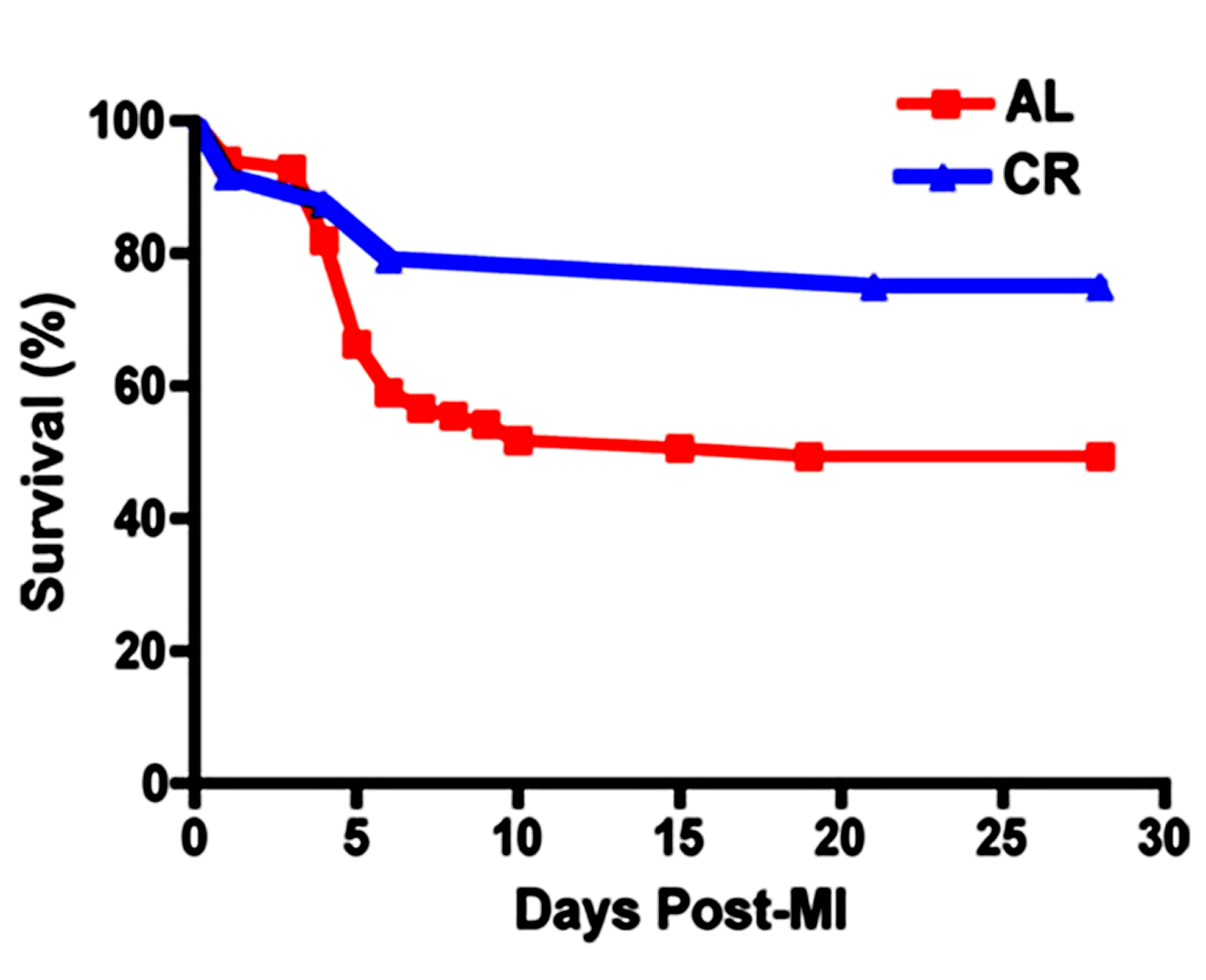

Supplement: S2 File — Fig. A. Survival post-MI was significantly higher in the CR mice compared to AL controls as demonstrated by Kaplan-Meier survival curves (N = 15, P = 0.001). Fig. B: qPCR validation of differentially regulated genes upregulated (Panel A) and downregulated (Panel B) genes in CR vs. AL from the DNA microarray analysis. The mRNA levels were normalized to the housekeeping gene Gapdh. Fig. C: Hierarchical clustering representation of the genes differentially expressed (genes upregulated or downregulated 1.2 fold in the heart samples of Liraglutide and CR group relative to Ad lib). The color gradient (red, up-regulation; bleu, down-regulation) depicts normalized gene expression of all of the samples. Panel A: the heat-map of gene expression depicts a more similar expression pattern in Liraglutide and AL conditions with an opposite pattern of expression in CR. Panel B: Principal Component Analysis (PCA) representation of replicate microarray gene expression profiles of heart samples from CR, Liraglutide, and AL mice (green, blue, and red symbols, respectively). PCA to visualize overall clustering of the microarray data showed that the transcriptional profiles were reproducible with more similar expression pattern in Liraglutide and Ad lib conditions and discrete from CR. Fig. D: Western blot assessment of LC3 and p-Akt/Akt expression. Protein abundance of autophagy marker LC3A/BII is significantly reduced (P = 0.001) in the LV of CR mice pre-MI (Panel A). AL and sAL regimens had no effect on the phosphorylation status of Akt, while CR resulted in increased phosho-Akt levels (Panel B). (ZIP) [file pone.0130658.s002.zip › S2_Fig._A.TIF]

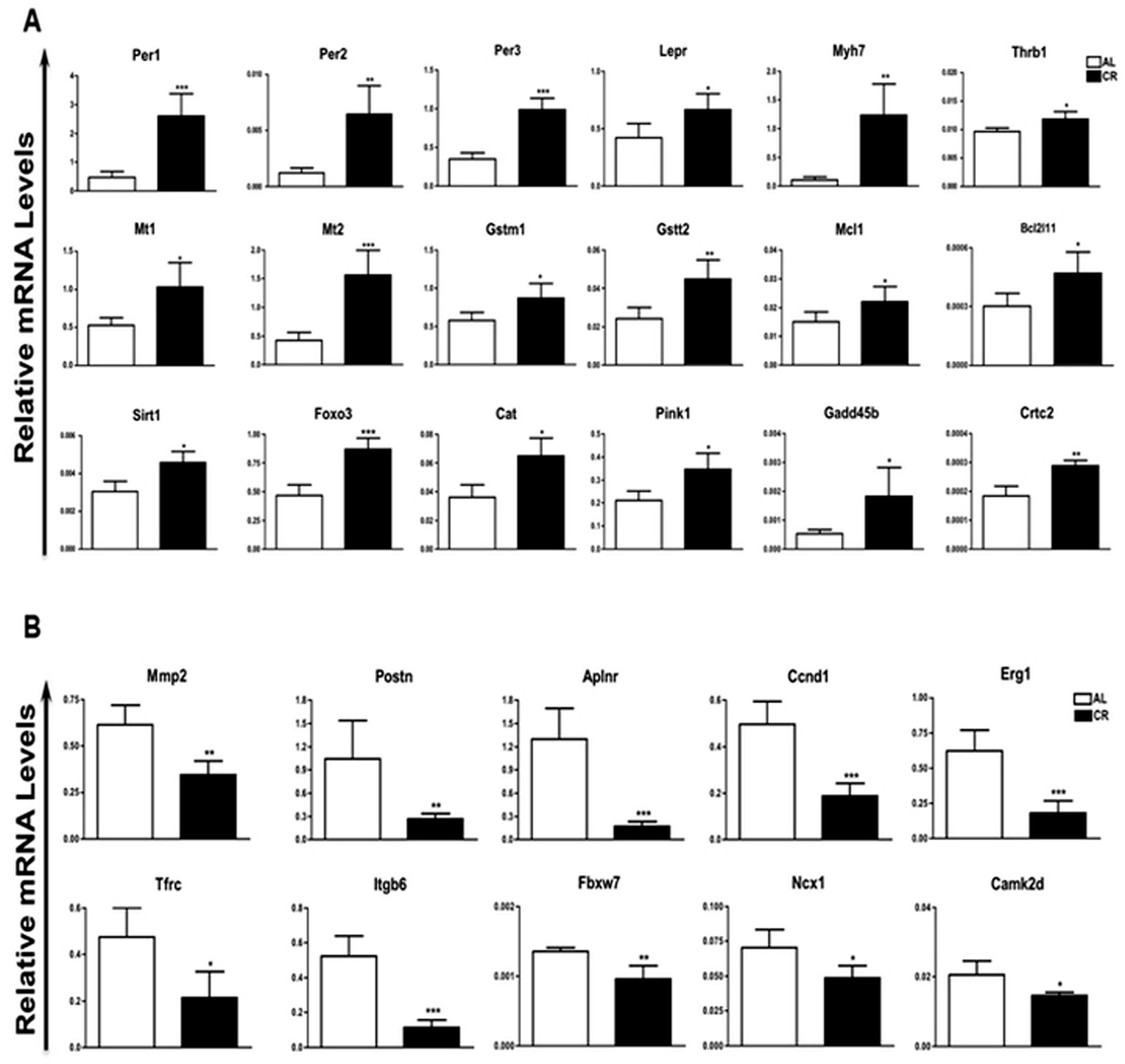

Supplement: S2 File — Fig. A. Survival post-MI was significantly higher in the CR mice compared to AL controls as demonstrated by Kaplan-Meier survival curves (N = 15, P = 0.001). Fig. B: qPCR validation of differentially regulated genes upregulated (Panel A) and downregulated (Panel B) genes in CR vs. AL from the DNA microarray analysis. The mRNA levels were normalized to the housekeeping gene Gapdh. Fig. C: Hierarchical clustering representation of the genes differentially expressed (genes upregulated or downregulated 1.2 fold in the heart samples of Liraglutide and CR group relative to Ad lib). The color gradient (red, up-regulation; bleu, down-regulation) depicts normalized gene expression of all of the samples. Panel A: the heat-map of gene expression depicts a more similar expression pattern in Liraglutide and AL conditions with an opposite pattern of expression in CR. Panel B: Principal Component Analysis (PCA) representation of replicate microarray gene expression profiles of heart samples from CR, Liraglutide, and AL mice (green, blue, and red symbols, respectively). PCA to visualize overall clustering of the microarray data showed that the transcriptional profiles were reproducible with more similar expression pattern in Liraglutide and Ad lib conditions and discrete from CR. Fig. D: Western blot assessment of LC3 and p-Akt/Akt expression. Protein abundance of autophagy marker LC3A/BII is significantly reduced (P = 0.001) in the LV of CR mice pre-MI (Panel A). AL and sAL regimens had no effect on the phosphorylation status of Akt, while CR resulted in increased phosho-Akt levels (Panel B). (ZIP) [file pone.0130658.s002.zip › S2_Fig._B.tif]

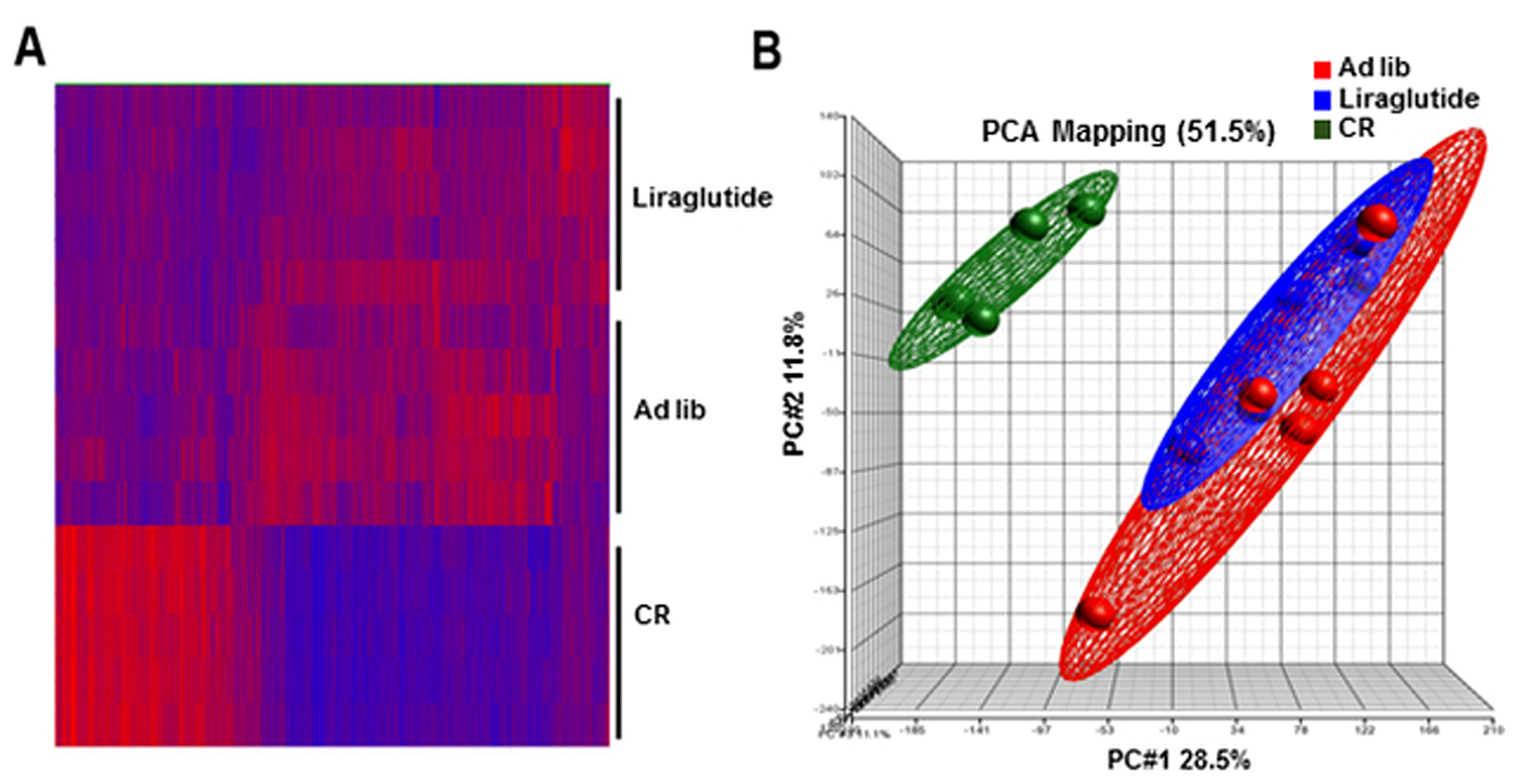

Supplement: S2 File — Fig. A. Survival post-MI was significantly higher in the CR mice compared to AL controls as demonstrated by Kaplan-Meier survival curves (N = 15, P = 0.001). Fig. B: qPCR validation of differentially regulated genes upregulated (Panel A) and downregulated (Panel B) genes in CR vs. AL from the DNA microarray analysis. The mRNA levels were normalized to the housekeeping gene Gapdh. Fig. C: Hierarchical clustering representation of the genes differentially expressed (genes upregulated or downregulated 1.2 fold in the heart samples of Liraglutide and CR group relative to Ad lib). The color gradient (red, up-regulation; bleu, down-regulation) depicts normalized gene expression of all of the samples. Panel A: the heat-map of gene expression depicts a more similar expression pattern in Liraglutide and AL conditions with an opposite pattern of expression in CR. Panel B: Principal Component Analysis (PCA) representation of replicate microarray gene expression profiles of heart samples from CR, Liraglutide, and AL mice (green, blue, and red symbols, respectively). PCA to visualize overall clustering of the microarray data showed that the transcriptional profiles were reproducible with more similar expression pattern in Liraglutide and Ad lib conditions and discrete from CR. Fig. D: Western blot assessment of LC3 and p-Akt/Akt expression. Protein abundance of autophagy marker LC3A/BII is significantly reduced (P = 0.001) in the LV of CR mice pre-MI (Panel A). AL and sAL regimens had no effect on the phosphorylation status of Akt, while CR resulted in increased phosho-Akt levels (Panel B). (ZIP) [file pone.0130658.s002.zip › S2_Fig._C.TIF]

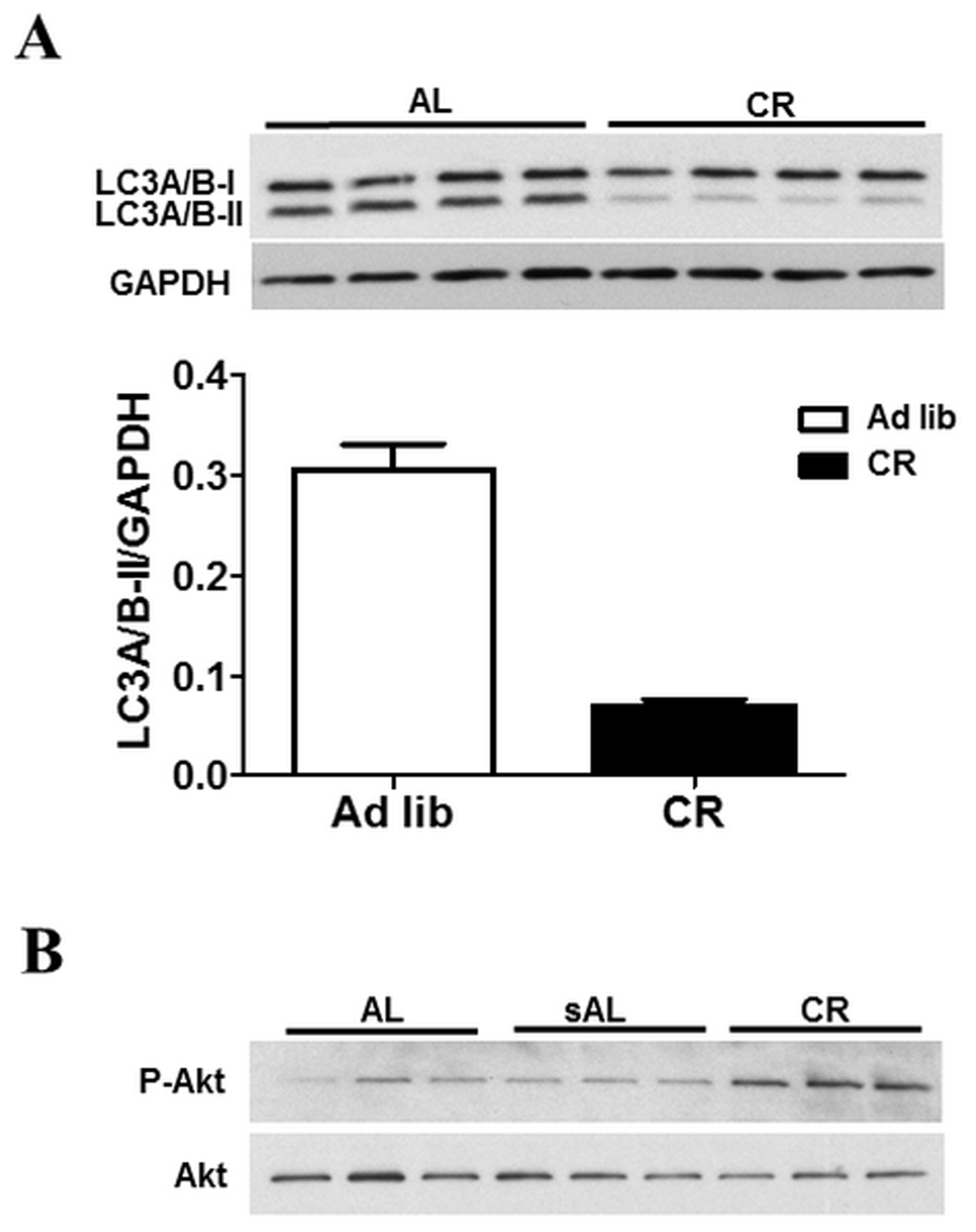

Supplement: S2 File — Fig. A. Survival post-MI was significantly higher in the CR mice compared to AL controls as demonstrated by Kaplan-Meier survival curves (N = 15, P = 0.001). Fig. B: qPCR validation of differentially regulated genes upregulated (Panel A) and downregulated (Panel B) genes in CR vs. AL from the DNA microarray analysis. The mRNA levels were normalized to the housekeeping gene Gapdh. Fig. C: Hierarchical clustering representation of the genes differentially expressed (genes upregulated or downregulated 1.2 fold in the heart samples of Liraglutide and CR group relative to Ad lib). The color gradient (red, up-regulation; bleu, down-regulation) depicts normalized gene expression of all of the samples. Panel A: the heat-map of gene expression depicts a more similar expression pattern in Liraglutide and AL conditions with an opposite pattern of expression in CR. Panel B: Principal Component Analysis (PCA) representation of replicate microarray gene expression profiles of heart samples from CR, Liraglutide, and AL mice (green, blue, and red symbols, respectively). PCA to visualize overall clustering of the microarray data showed that the transcriptional profiles were reproducible with more similar expression pattern in Liraglutide and Ad lib conditions and discrete from CR. Fig. D: Western blot assessment of LC3 and p-Akt/Akt expression. Protein abundance of autophagy marker LC3A/BII is significantly reduced (P = 0.001) in the LV of CR mice pre-MI (Panel A). AL and sAL regimens had no effect on the phosphorylation status of Akt, while CR resulted in increased phosho-Akt levels (Panel B). (ZIP) [file pone.0130658.s002.zip › S2_Fig._D.TIF]
